# Platelet-rich plasma as an adjuvant therapy for intrauterine adhesions: a narrative review of mechanisms, clinical efficacy and combination strategies

**DOI:** 10.1080/07853890.2026.2731715

**Published:** 2026-09-17

**Authors:** Dandan Xiong, Guangli Mao, Jianwu Zhao, Dan Zi, Fang Qiu

**Affiliations:** a Department of Obstetrics and Gynecology, Zunyi Medical University, Guizhou, China; b Department of Obstetrics and Gynecology, Guizhou Provincial People’s Hospital, Guizhou, China

**Keywords:** Intrauterine adhesions, endometrium, platelet-rich plasma, combined therapy, reproductive outcomes

## Abstract

**Background:**

Intrauterine adhesions (IUA), particularly moderate to severe cases, is a leading cause of decreased menstrual flow and secondary infertility in women, affecting an estimated 2.8%∼46% of infertile women. Hysteroscopy remains the gold standard for diagnosis, with high sensitivity and specificity (both exceeding 90%), while three-dimensional ultrasonography offers a non-invasive alternative with a reported sensitivity of 86% and specificity of 90% for detecting intrauterine abnormalities. The success of surgical management *via* transcervical resection of adhesions (TCRA) is primarily gauged by the resumption of normal menstrual flow and the absence of significant adhesions on second-look hysteroscopy or ultrasonography. However, the high recurrence rate postoperatively, especially in severe cases (up to 60%), and the persistently thin endometrium significantly impair patients’ reproductive outcomes. Existing adjuvant treatments such as high-dose oestrogen, uterine stents and hydrogels have failed to substantially improve patients’ fertility outcomes. Platelet-rich plasma (PRP), as an autologous biological preparation rich in various growth factors, has demonstrated remarkable application prospects in recent years for the adjuvant and combined treatment of IUA due to its potent pro-angiogenic, anti-inflammatory and tissue repair/regeneration-promoting properties.

**Objective:**

This review aims to summarize the mechanisms and clinical efficacy of PRP as an adjuvant therapy for IUA, providing new perspectives for the comprehensive management of this condition.

**Discussion:**

We explore the mechanisms of action and clinical outcomes of PRP monotherapy following IUA surgery and evaluate the safety and application advantages of PRP and autologous PRP. The mechanisms of the synergistic effects of PRP combined with mesenchymal stem cells(MSCs), uterine scaffolds and hydrogels, as well as the efficacy of these combination therapies, were discussed. In most studies, favourable feedback was obtained, while a few studies suggested that PRP alone or in combination failed to improve the reproductive outcomes of IUA patients.

**Conclusion:**

Current evidence suggests that PRP, both as a standalone and adjunctive therapy, shows promise in promoting endometrial regeneration and improving certain clinical outcomes, such as endometrial thickness and menstrual flow, in patients with IUA. However, the clinical efficacy remains inconclusive due to significant heterogeneity in study designs, patient populations and PRP preparation protocols. High-quality, large-scale randomized controlled trials with standardized methodologies and long-term follow-up are urgently needed to definitively establish its impact on critical reproductive outcomes, such as live birth rates, before its widespread clinical adoption can be recommended.

## 1. Introduction

Intrauterine adhesions (IUA), commonly referred to as Asherman’s syndrome (AS), are pathologically identified by fibrosis that results from damage to the endometrium’s basal layer. During the healing stage of the endometrium, either thin or dense fibrous bands may develop, which can cause partial or complete blockage of the uterine cavity [1]. The clinical presentation of IUA predominantly consist of menstrual irregularities, amenorrhoea, infertility and recurring miscarriages. These manifestations critically hinder female reproductive functionalities, affecting both reproductive health and psychological wellbeing [2]. Among these symptoms, infertility poses a particularly severe challenge, ranking as one of the most significant clinical issues. At present, hysteroscopy is regarded as the gold standard for diagnosing IUA and assessing the effectiveness of postoperative interventions. This procedure allows for direct visualization of the uterine cavity’s structure, including the locations and extent of adhesions, as well as any scarring. The preferred therapeutic method is transcervical resection of adhesions (TCRA), which aims to restore the uterine cavity to its typical size and shape, thus facilitating the recovery of endometrial function [3]. Nevertheless, in cases with severe endometrial fibrosis, the recurrence rate for IUA following surgery can be as high as 60% [4]. Despite the administration of routine high-dose oestrogen therapy after surgery, outcomes frequently fall short of expectations [5,6]. Clinically, a recurring cycle of adhesion formation, separation and re-adhesion is often encountered, which suggests that current treatment options have limited effectiveness in enhancing patients’ quality of life and reproductive success, with notable negative implications on their physical and mental health. Consequently, exploring effective therapeutic approaches to enhance patients’ daily quality of life and reproductive outcomes holds considerable clinical significance.

Platelet-rich plasma (PRP) is an innovative adjunctive treatment for tissue regeneration and repair, offering benefits such as straightforward separation, non-invasive application and the ability to use the patient’s own blood [7]. It has seen extensive use across various medical fields, including maxillofacial surgery, plastic surgery, orthopaedics, dermatology, urology and gynaecology [8]. PRP is derived from fresh whole blood collected from peripheral veins and is processed to create platelet concentrations that are 4 to 8 times greater than those found in whole blood, often exceeding 1,000,000 platelets per microlitre [9]. Recently, intrauterine infusion of PRP has been utilized as a supplemental therapy during adhesiolysis procedures to enhance clinical outcomes for patients with IUA [10]. Administering PRP at the injury location releases numerous bioactive agents, such as platelet-derived growth factors (PDGFs), transforming growth factors (TGFs), insulin-like growth factors (IGFs), vascular endothelial growth factor (VEGF), epidermal growth factor (EGF) and fibroblast growth factors (FGFs). These bioactive agents stimulate cell migration and localization to the injury site while facilitating cell proliferation, differentiation and angiogenesis [11,12]. The factors derived from platelets play a vital role in the function of endometrial progenitor cells [13], with different PDGF subtypes significantly augmenting proliferation, migration and contractile ability in endometrial stromal cells [14–16]. Studies show that PRP can enhance menstrual flow volume, extend menstrual duration, increase endometrial thickness and raise clinical pregnancy rates [17]. Nonetheless, the effectiveness of PRP in treating patients with IUA is still a matter of debate, highlighting the need for more evidence-based research in this area [18,19]. With continuous advances in biotechnology and in-depth preclinical model studies, a solid foundation has been laid for developing personalized treatment protocols for infertility-related endometrial disorders such as IUA.

Although there is currently a lack of independent, specific statistical data on PRP as an adjunctive therapy for intrauterine adhesions, existing high-quality Meta-analyses and ongoing clinical studies indicate that PRP is a cutting-edge technology with high-level evidence supporting its overall effectiveness, which is currently undergoing targeted validation. This review comprehensively summarises the current status of PRP application in the adjunctive and combined treatment of IUA, encompassing its mechanisms of action, clinical efficacy and safety assessment. It further explores the clinical value and future development directions of PRP therapy for IUA.

## 2. Methods

### 2.1. Data sources and literature search

Two databases, PubMed and Web of Science, were searched for all relevant articles published between 2015 and 2025, including basic science (*in vitro* and animal experiments), observational studies and randomized studies. During the literature search, a broad range of keywords were used, such as “platelet-rich plasma, autologous platelet-rich plasma, intrauterine adhesions, Asherman’s syndrome, thin endometrium, endometrium, aetiology, pathogenesis, clinical manifestations, menstrual volume, pregnancy rate, mechanism of action, endometrial proliferation, fibrosis, angiogenesis, angiogenesis mechanism, inflammation, endometrial mesenchymal stem cells, mesenchymal stem cells, stem cells, safety, preparation methods and application advantages.” Use MeSH(Medical Subject Headings) terms when searching on PubMed.

### 2.2. Inclusion and exclusion criteria

All studies meeting the following criteria were eligible for inclusion in this review: Firstly, basic research and clinical studies involving PRP and/or intrauterine adhesions, PRP and/or thin endometrium; Secondly, reporting at least one of our desired outcomes, including: clinical pregnancy rate, changes in endometrial thickness, menstrual flow, duration of menstruation, adhesion recurrence, reduction in American Fertility Society (AFS) scores. Reviews, short communications, letters to the editor and other studies that did not meet the above criteria were excluded from this review. Consequently, 45 studies were ultimately identified and included.

## 3. Mechanisms and efficacy of PRP monotherapy in IUA treatment

### 3.1. Mechanistic basis

#### 3.1.1. Promotion of endometrial cell proliferation and inhibition of fibrosis

The endometrium constitutes the inner lining of the uterus, primarily composed of stroma and glands and is crucial for successful embryo implantation and maintaining normal uterine function [20,21]. Endometrial tissue undergoes cyclical shedding, repair and regeneration throughout the menstrual cycle. These physiological processes are characterized by the regulation of hormones, cellular proliferation, decidualization, inflammatory reactions, episodes of hypoxia, programmed cell death (apoptosis), the regulation of blood clotting (haemostasis) and vasoconstriction [20]. Extensive damage to the endometrium can result in a thinner endometrial lining or IUA, both of which are associated with a poor prognosis. Impaired of the endometrium leads to degenerative alterations, including narrowing of the uterine cavity, atrophy of columnar epithelial cells, a reduction in the number of endometrial glands, a decrease in the thickness of the endometrium and an increase in collagen accumulation [9,22]. The endometrium consists of a functional layer, which undergoes cyclic shedding during menstruation and a basal layer responsible for regeneration. In IUA, damage to the basal layer – often resulting from curettage or infection – disrupts this regenerative capacity, leading to fibrous tissue deposition and intrauterine scar formation. During the healing stage of the endometrium, the imbalance between fibrinogenesis and fibrinolysis promotes excessive extracellular matrix accumulation. This pathological remodelling impairs endometrial receptivity and vascularization, ultimately compromising fertility outcomes [23]. Thus, while normal endometrial physiology provides the context, it is the disruption of this delicate system that underlies IUA pathogenesis. Recent studies have shown that PRP may enhance endometrial hyperplasia, although the exact mechanisms of regulation remain elusive. Mao et al. explored the therapeutic effects and underlying mechanisms of PRP in promoting endometrial regeneration [22]. After creating a model of IUA, PRP was injected bilaterally into the uterine horns of rats. When compared to the model group, notable increases were found in endometrial thickness, the number of endometrial glands and the area of the endometrium, while collagen deposition showed a decrease. This indicates PRP promotes endometrial regeneration and reduces fibrosis.

#### 3.1.2. Regulation of proliferation and migration in endometrial mesenchymal stem/stromal cells (EndoMSCs)

Endometrial mesenchymal stem/stromal cells (EndoMSCs) constitute a crucial cellular component responsible for extracellular matrix(ECM) remodelling, angiogenesis, intercellular communication and post-menstrual tissue repair, playing a pivotal role in endometrial tissue homeostasis [24,25]. Vishnyakova et al. conducted a comparison of the impacts of autologous PRP, autologous ordinary plasma (OP) and a complete growth medium on mesenchymal stem cells (MSCs) derived from rat uterine tissue [12]. The assessment of cellular proliferation using immunohistochemical staining for Ki-67 demonstrated a proliferation index of 41.9% within the PRP group (*p* = 0.04). Additionally, the research highlighted a statistically significant elevation in the expression of LC3B protein, which serves as an autophagy marker, in MSCs after exposure to PRP. These results suggest that PRP not only fosters the proliferation of uterine MSCs but also boosts the self-renewal capacity of endometrial stromal cells by enhancing autophagy. In the investigation conducted by de Miguel-Gomez et al. the effects of PRP obtained from various sources (human peripheral venous blood and human umbilical cord) on the proliferation and migration of human endometrial stromal cells *in vitro* were evaluated [7]. Both sources notably facilitated cell proliferation (activated human PRP *p* < 0.0394; activated cord blood plasma *p* < 0.0001). The cord blood-derived plasma demonstrated superior cell proliferation-promoting effects. Furthermore, the study contrasted activated PRP with activated platelet-depleted plasma, revealing that activated PRP exhibited greater potential for stimulating cell proliferation (*p* < 0.0034). KEGG pathway enrichment analysis detected shared PI3K-Akt signalling pathways in both human whole blood and umbilical cord plasma. In summary, PRP promotes the migration and proliferation of EndoMSCs, subsequently facilitating their differentiation into endometrial cells. In summary, PRP elevates the treatment of intrauterine adhesions from mere ‘mechanical adhesion separation’ to a new level of ‘functional endometrial regeneration’ by precisely activating and enhancing the patient’s own EndoMSCs to combat fibrosis and promote angiogenesis.

PDGFs represent a significant category of growth factors found in PRP. Among them, PDGF-BB stands out as the most potent subtype, triggering various signalling pathways that facilitate cell migration, survival and growth. Zhang et al. explored the anti-fibrotic properties of exosomes derived from menstrual blood-derived mesenchymal cells that had been pretreated with PDGF-BB (EXOPDGFBB) on *in vitro* IUA-EndoMSCs [26]. In the comparison between IUA-EndoMSCs and normal EndoMSCs, the levels of fibrosis-related proteins such as YAP, collagen I, CTGF and SMAD3 were found to be significantly higher in the former. The findings from Western blot analysis indicated that YAP expression in the EXOPDGFBB treatment group was notably lower than that in the conventional exosome group (*p* < 0.01). After treatment with EXOPDGFBB, IUA-EndoMSCs showed an increase in YAP ubiquitination, which led to a decrease in YAP levels and a reduction in its interaction with SMAD3, ultimately resulting in less fibrosis in these cells. It has been shown that excessive YAP activation is critical in the pathological mechanisms associated with cardiac, pulmonary, hepatic, renal and cutaneous fibrosis [27]. From a clinical perspective, the PDGF-BB-mediated activation of endometrial stem cells and vascular regeneration underscores its therapeutic potential in restoring endometrial function. This mechanistic insight supports the use of PRP as a biologic therapy aimed not merely at symptomatic improvement, but at promoting structural and functional repair in damaged endometrial tissue-an outcome particularly relevant for IUA patients with poor response to conventional treatments.

#### 3.1.3. Promotion of angiogenesis

**Figure 1.**
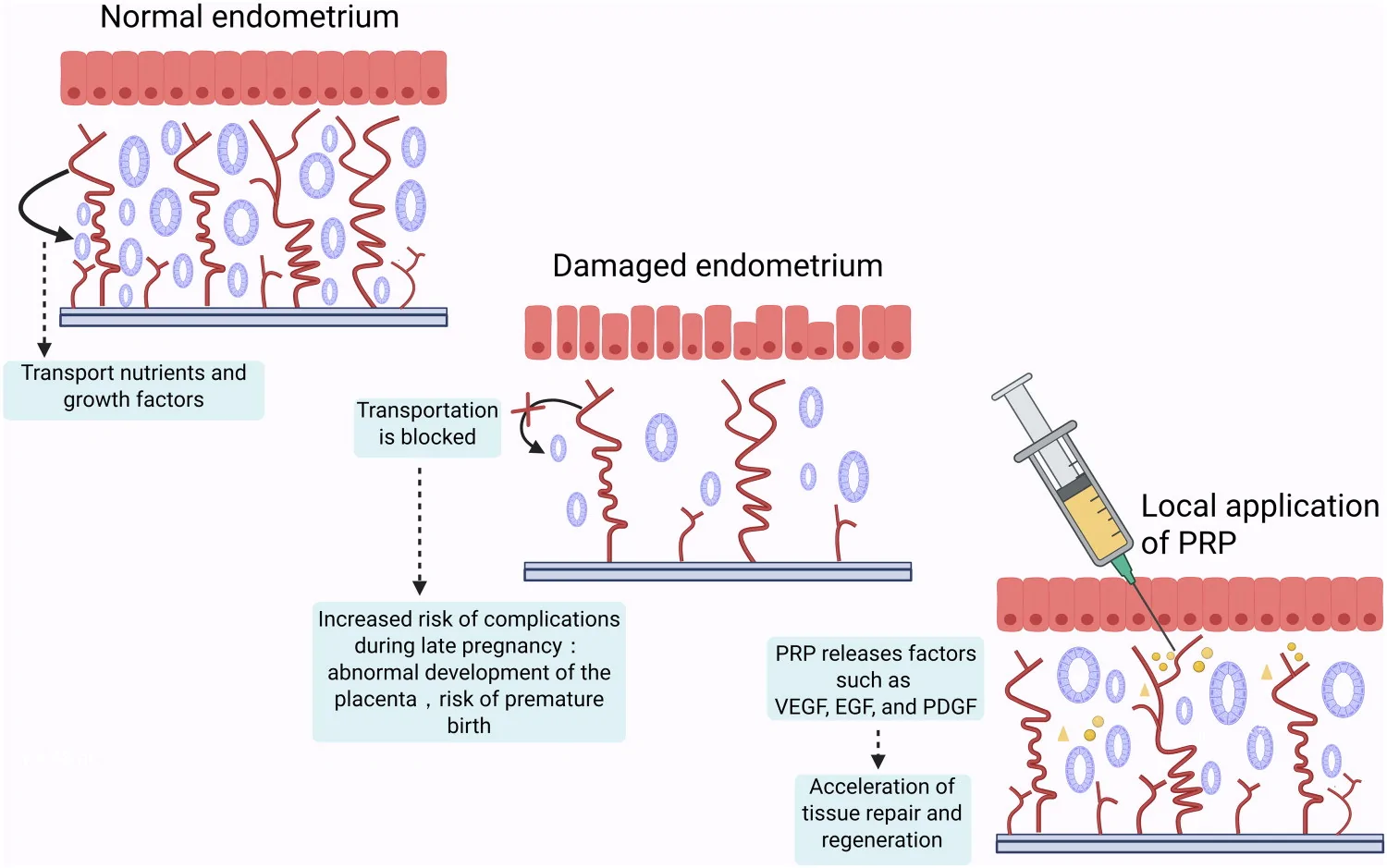
Schematic diagram of the mechanism by which PRP repairs endometrial dysfunction after IUA surgery through promoting angiogenesis.

The formation of new blood vessels facilitates the transport of nutrients and growth factors to cells that are dividing, which is essential for the processes of tissue repair and regeneration. Following IUA resection, impaired endometrial angiogenesis and metabolic function persist, leaving patients at risk of placental developmental abnormalities and preterm birth [28]. PRP is known to contain multiple growth factors that accelerate angiogenesis, thereby promoting rapid tissue repair and regeneration (Figure 1). In a study by Kim et al. real-time quantitative RT-PCR analysis of endometrial tissue from mice with AS revealed that human PRP treatment significantly upregulated mRNA expression levels of pro-angiogenic factors, including Hif1α, Hif2α, Vegf-a, Ang-1, Hgf and Igf-1 [29]. Furthermore, Western blotting analysis of pro-angiogenic factors at the protein level further corroborated the pro-angiogenic effect of human PRP plasma in AS uterine tissue. Among the various markers associated with angiogenesis, both HIF1α and HIF2α play a crucial role in inducing VEGF, which is responsible for regulating the angiogenesis switch, as well as vascular permeability and the migration and proliferation of endothelial cells [30]. The vasculogenic capabilities of PRP therapy were additionally supported by Zhang et al. [31], especially when sourced from human umbilical cord blood [32]. Cheng et al. explored the impact of combining extracorporeal shock wave therapy with PRP during both preventive and treatment stages in a rat model of IUA [33]. This research focused specifically on the effects of intrauterine PRP injection alone on angiogenesis. During the prevention phase, intrauterine PRP injection alone significantly upregulated VEGF mRNA and protein expression levels compared to the IUA injury group. During the therapeutic phase, it also markedly increased VEGF mRNA and protein expression, indicating that PRP may participate in the repair process of the IUA-damaged uterine cavity by promoting angiogenesis.

This diagram illustration highlights the vital importance of angiogenesis in the healing of the endometrium, the long-term risks caused by impaired angiogenesis after IUA surgery and the potential therapeutic mechanism of PRP in promoting angiogenesis by providing key growth factors, thereby reversing dysfunction and facilitating regeneration.

#### 3.1.4. Modulation of inflammatory responses

Endometrial receptivity significantly relies on the inflammatory status and the presence of favourable antimicrobial conditions. Research has validated the anti-inflammatory properties of PRP. Reghini et al. showcased notable anti-inflammatory effects of PRP on the endometrium of mares [34]. After administering intrauterine PRP injections to mares suffering from chronic endometritis, substantial decreases in neutrophil infiltration and the volume of uterine cavity effusion were recorded 24 h following treatment. To delve deeper into the anti-inflammatory properties of PRP, Marini et al. utilized an *in vitro* model that simulates endometrial cell inflammation through the application of 10 ng/ml bacterial endotoxin lipopolysaccharide(LPS) [35]. Upon LPS exposure, the expression of interleukin-1β(IL-1β), inducible nitric oxide synthase (iNOS), cyclooxygenase-2 (COX2) and interleukin-8(IL-8) significantly increased when compared to untreated cells (*p* < 0.01). The application of PRP notably diminished the expression levels of these genes in the endometrium(*p* < 0.01), highlighting the therapeutic promise of PRP for regenerative treatments of endometritis. In the ethanol-induced endometrial injury model developed by Jang et al. significant upregulation of the pro-inflammatory cytokine IL-1β, as well as the anti-inflammatory cytokine interleukin-10 (IL-10), was observed, indicating that the formation of IUA is linked to inflammatory responses [9]. The mRNA expression of the pro-inflammatory cytokine IL-1βwas considerably lower in the group treated with PRP compared to the ethanol-treated group, demonstrating the anti-inflammatory effectiveness of PRP.

Previous studies have clarified how PRP facilitates endometrial repair and addresses IUA through various mechanisms. The primary mechanism involves the collaborative enhancement of endometrial cell and MSC proliferation and migration, attributed to its abundant growth factors, while also suppressing fibrosis-related pathways and modulating pathways associated with cell death. Concurrently, PRP enhances angiogenesis by upregulating pro-angiogenic factors while exerting potent anti-inflammatory effects. Collectively, these actions reshape a regenerative microenvironment, ultimately restoring endometrial structure and function. However, existing evidence primarily derives from animal models and *in vitro* experiments, with relatively scarce clinical data. Optimal therapeutic dosages and regimens remain unstandardized. Future studies require extensive, multicentric randomized controlled trials to confirm the long-term effectiveness and safety, as well as a thorough investigation of its particular molecular signalling pathways.

### 3.2. Clinical efficacy assessment

To date, PRP therapy has been applied for over 30 years across multiple fields including orthopaedics, dermatology and dentistry, yielding notable outcomes. Although its application in obstetrics and gynaecology remains exploratory, it has demonstrated considerable efficacy. A retrospective analysis demonstrated that the infusion of PRP into the uterus after hysteroscopic adhesiolysis significantly decreased adhesion scores, proposing PRP as a possible therapy for IUA [36]. Furthermore, a randomized controlled trial confirmed that intrauterine infusion of PRP following IUA surgery significantly reduced postoperative adhesion formation. Compared to patients not receiving PRP treatment, those treated with PRP demonstrated marked improvements in menstrual flow and endometrial thickness [37]. However, it remains unclear whether these improvements are directly attributable to PRP therapy, and the current lack of robust research evidence may be linked to the absence of objective efficacy measures. Recent non-randomized clinical trial findings by Javaheri et al. challenge these conclusions [38]. This research involved 30 individuals with a background of AS, all of whom underwent hysteroscopic adhesiolysis followed by the placement of an intrauterine catheter prior to being randomly assigned to either the PRP group or a control group. Each participant had a follow-up diagnostic hysteroscopy conducted 8–10 weeks after the intervention. The findings indicated no significant differences in hysteroscopic evaluations of IUA or menstrual cycle patterns between the groups; however, pregnancy outcomes were not assessed. The effectiveness of autologous PRP therapy is still debated – some researchers reported an increase in endometrial thickness [39–41], whereas others found no such effect [42,43]. This inconsistency might be affected by the method of administration [39], processing methods, as well as the age of patients and any existing comorbidities [44]. Currently, there is a lack of comprehensive data regarding the ideal PRP dosage, frequency of treatments and the long-term safety and effectiveness of the therapy. Additionally, the absence of standardized methods for preparation and application hampers the ability to compare results across different studies. The specific clinical efficacy data of relevant studies on PRP monotherapy for IUA, TE and RIF are summarized in Table 1. A comprehensive summary of clinical studies on autologous PRP monotherapy for IUA and its associated endometrial pathologies is shown in Table 2.

**Table 1.** Summary of clinical efficacy comparisons.

Study ID	Study design	IUA/AS/TE/RIF	Sample size	PRP		Outcome measure	Main discovery
				Autologous/allogeneic	Dose		
Peng et al. [36]	Retrospective	Moderate to severe IUA	70	Autologous	0.5–1 ml	1. AFS score 2. Rates of chemical pregnancy	None were statistically significant
Shen et al. [37]	RCT	Moderate to severe IUA	123	Autologous	4 ml	1. AFS score 2. PBAC score 3. Endometrial thickness	All were statistically significant
Javaheri et al. [38]	Non-RCT	AS	30	Autologous	1 ml	1. Menstrual pattern 2. Intrauterine adhesion stage 3. Menstrual bleeding duration	None were statistically significant
Dogra et al. [39]	Prospective interventional cohort study	Refractory TE (<7mm)	20	Autologous	0.5–1 ml	1. Endometrial thickness 2. Live birth rates 3. Implantation rate 4. Clinical pregnancy rate	Mean EMT increased
Tandulwadkar et al. [40]	Not specified	Poor endometrial growth, multiple cycle cancellations	68	Autologous	0.5–0.8 ml	1. Endometrial thickness 2. Implantation rate, clinical pregnancy rate and outcome of pregnancy	EMT increased
Chang et al. [41]	Prospective cohort study	TE	64	Autologous	0.5–1 ml	1. Endometrial thickness 2. Cycle cancellation rate 3. Implantation rate and clinical pregnancy rate	All were statistically significant
Aghajanova et al. [42]	Phase II pilot randomized controlled single blinded study (RCT)	Moderate to severe AS	30	Autologous	0.5–1 ml	1. Endometrial thickness 2. Menstrual bleeding pattern 3. Pregnancy rate	None were statistically significant
Enatsu et al. [43]	Retrospective	TE, unexplained implantation failure	54	Autologous	1 ml	1. Endometrial thickness 2. HCG positivity rate 3. Clinical pregnancy	Improve HCG positive rate and clinical pregnancy

*Note:* RIF: repeated implantation failure; TE: thin endometrium; RCT: randomized controlled trial; PBAC: Pictorial Blood-loss Assessment Chart; AFS: American Fertility Society; HCG: Human Chorionic Gonadotropin.

**Table 2.** Summary of clinical studies on platelet-rich plasma monotherapy.

Study ID	Country	Study design	Population	Sample size	Outcome measure	Main discovery
Javaheri et al. [38]	Iran	Non-RCT	AS	PRP: 15 Did not receive PRP: 15	1. Menstrual pattern 2. Intrauterine adhesion stage 3. Menstrual bleeding duration	None were statistically significant
Dogra et al. [39]	India	Prospective interventional cohort study	Refractory TE (<7mm)	PRP: 20 (before-after comparison)	1. Endometrial thickness 2. Live birth rates 3. Implantation rate 4. Clinical pregnancy rate	Mean EMT increased
Tandulwadkar et al. [40]	India	A pilot study	Poor endometrial growth, multiple cycle cancellations	PRP: 68 (before-after comparison)	1. Endometrial thickness 2. Implantation rate, clinical pregnancy rate and outcome of pregnancy	EMT increased
Chang et al. [41]	China	Prospective cohort study	TE	PRP:34 Did not receive PRP: 30	1.Endometrial thickness 2. Cycle cancellation rate 3. Implantation rate and clinical pregnancy rate	All were statistically significant
Aghajanova et al. [42]	America	Phase II pilot randomized controlled single blinded study (RCT)	Moderate to severe AS	PRP: 15 Normal saline: 15	1. Endometrial thickness 2. Menstrual bleeding pattern 3. Pregnancy rate	None were statistically significant
Enatsu et al. [43]	Japan	Retrospective	TE, unexplained implantation failure	TE: 39 Unexplained implantation failure: 15 Both groups received PRP treatment (before-after comparison)	1. Endometrial thickness 2. HCG positivity rate 3. Clinical pregnancy	Improve HCG positive rate and clinical pregnancy
Kim et al. [46]	Korea	Interventional prospective cohort study	Refractory TE	PRP: 20 (before-after comparison)	1. Continuous pregnancy rate 2. Live birth rate 3. Endometrial thickness 4. Implantation rate 5. Clinical pregnancy rate	Improve implantation rate and clinical pregnancy rate
Nazari et al. [49]	Iran	Preliminary single-arm study of a randomized controlled trial	RIF	PRP:20 (before-after comparison)	Pregnancy rate	18 cases of pregnancy: Sixteen clinical pregnancies One early miscarriage One molar pregnancy
Zamaniyan et al. [50]	Iran	RCT	RIF	Intervention group: 55 Control group: 43	1. Endometrial thickness 2. Clinical pregnancy rate 3. Sustained pregnancy rate 4. Embryo implantation rate	All were statistically significant
Allahveisi et al. [52]	Iran	RCT	RIF	PRP: 25 Ringer’s serum: 25	1. Chemical pregnancy rate 2. Clinical pregnancy rate	None were statistically significant
Fan et al. [53]	China	Retrospective cohort study	Severe IUA	PRP: 25 Control group: 25	1. Thickness of endometrium and blood flow 2. Pregnancy rate	None were statistically significant

*Note:* RIF: repeated implantation failure; TE: thin endometrium; RCT: randomized controlled trial; HCG: Human Chorionic Gonadotropin.

### 3.3. Improving pregnancy outcomes

In 2018, Aghajanova et al. were the first to document the clinical utilization of PRP in the treatment of AS [45]. Their reports on two cases illustrated that PRP enhanced endometrial function and aided in achieving successful pregnancies among patients with AS. Research has indicated that PRP therapy can augment endometrial thickness (as measured *via* ultrasound), boost clinical pregnancy rates and improve overall pregnancy outcomes [46]. Kim et al. performed a quantitative assessment of pregnancy and implantation results, uncovering that PRP interventions significantly elevated the number of implantation sites (IS) and supported the maintenance of pregnancies to full term [47]. The live birth rate observed in AS mice treated with PRP was 83.3%, in stark contrast to untreated AS mice, none of whom achieved successful deliveries. Puente Gonzalo et al. documented an instance involving a 31-year-old woman with a prior diagnosis of AS who continued to experience insufficient endometrial thickness despite hysteroscopic intervention [48]. After several unsuccessful attempts at endometrial enhancement therapies, the patient received intrauterine PRP injections (two doses administered three days apart). Post-PRP treatment, the endometrial thickness improved to 7.7 mm. A high-quality Grade A blastocyst was subsequently transferred, leading to a successful pregnancy outcome. The patient delivered a healthy infant at 37 weeks of gestation without encountering any complications. Numerous studies suggest that PRP considerably enhances results for patients facing recurrent implantation failure. For example, Nazari et al. reported a pregnancy rate of 90% [49], while a randomized controlled trial by Zamaniyan showed that the PRP cohort achieved notably higher clinical pregnancy rates, ongoing pregnancy rates and embryo implantation rates compared to the control cohort [50]. A comprehensive meta-analysis involving 625 patients provided strong evidence for the effectiveness of PRP, indicating significantly elevated biochemical pregnancy rates, clinical pregnancy rates and embryo implantation rates in patients treated with PRP relative to controls [51]. However, a randomized controlled trial by Allahveisi et al. found no significant difference in biochemical or clinical pregnancy rates between the PRP and control groups [52]. In a retrospective cohort study conducted by Fan et al. the intrauterine infusion of PRP did not lead to a significant enhancement in endometrial thickness or improvement in pregnancy rates among patients with severe IUA who underwent a follow-up hysteroscopy that showed a thin endometrium following surgery [53]. While recent research has raised doubts regarding the effectiveness of PRP as supplementary treatment for IUA, it is crucial that more comprehensive studies are carried out in this area. A detailed comparison of pregnancy rate outcomes in PRP intervention studies for different endometrial disorders is presented in Table 3.

**Table 3.** Summary of pregnancy rate comparison.

Study ID	Study design	IUA/TE/RIF	Sample size	PRP		Pregnancy rate
				Autologous/allogeneic	Dose	
Kim et al. [46]	Interventional prospective cohort study	Refractory TE	20	Autologous	0.7–1.0 ml	Implantation rate: 12.7%vs0% (*p* = .015) Clinical pregnancy rate: 30%vs0% (*p* = .02) Live birth rate: 20%vs0% (*p* = .106)
Nazari et al. [49]	Preliminary single-arm study of a randomized controlled trial	RIF	20	Autologous	0.5 ml	18 cases of pregnancy: Sixteen clinical pregnancies One early miscarriage One molar pregnancy
Zamaniyan et al. [50]	RCT	RIF	98	Autologous	0.5 ml	Clinical pregnancy: (48.3% vs 23.26%) (*p* = .001) Ongoing pregnancy (46.7% vs 11.7%) (*p* = .001) Implantation rate (58.3% vs 25%) (*p* = .001)
Allahveisi et al. [52]	RCT	RIF	50	Autologous	0.5 ml	Rate of chemical pregnancy (28% vs 36%) Clinical pregnancy (28% vs 24%) Live birth (24% vs 28%) No statistical significance
Fan et al. [53]	Retrospective cohort study	Severe IUA	50	Autologous	1.0 ml	Total pregnancy rate No statistical significance

*Note:* RIF: repeated implantation failure; TE: thin endometrium; RCT: randomized controlled trial.

The discrepancy in study results regarding PRP’s significant improvement in pregnancy rates versus negative conclusions may be attributed to several key differences. Variations in patient inclusion criteria serve as the primary factor contributing to divergent findings. Significant differences exist across studies in defining the ‘PRP-eligible population’, directly impacting efficacy evaluations. Some studies have conflated patients with intrauterine adhesions post-surgery and those with thin endometrium [53]. These two conditions differ fundamentally in pathological mechanisms – intrauterine adhesions primarily involve fibrotic repair, while thin endometrium may stem from various causes such as blood flow disorders or hormonal resistance. Pooling patients with distinct aetiologies in the same analysis dilutes the true therapeutic effect of PRP. Studies have confirmed that the postoperative pregnancy rate in patients with mild to moderate intrauterine adhesions is significantly higher than in those with severe adhesions and the degree of adhesion is an independent risk factor affecting reproductive outcomes [54]. Some studies included patients undergoing initial treatment, while others enrolled ‘refractory’ cases who had failed conventional therapies. There are substantial differences in patient inclusion criteria across studies, resulting in poor comparability. Another key factor contributing to inconsistent findings is the variability in PRP preparation. PRP is not a standardized pharmaceutical product but rather an autologous biological preparation, with significant variations in both the preparation process and final product among different studies and centres. Even though the dosage of PRP showed no significant differences in the aforementioned studies, there were considerable variations in centrifugation protocols and activation methods for PRP preparation across different studies. The routes of PRP administration, frequency and timing of administration, as well as intrauterine retention time of PRP also vary. Finally, there are differences in observation windows. Short-term follow-up may underestimate its long-term value, while long-term follow-up may better reflect the true clinical value of PRP, but it also introduces more confounding factors, such as patients’ subsequent treatment plans and chances of natural conception. Most studies use clinical pregnancy rate as the primary endpoint, but the definition of ‘clinical pregnancy’ (e.g. the timing of gestational sac appearance) may differ across studies. In summary, whether in adjuvant treatment after intrauterine adhesiolysis or in the treatment of thin endometrium or recurrent implantation failure, PRP therapy lacks standardized protocols, which is the main factor contributing to the variability in results among different studies.

Thoroughly structured randomized controlled trials are critically necessary to assess the effects of intrauterine PRP infusion on clinical outcomes, including the prevention of recurrent IUA, rates of pregnancy and live birth rates. Although the current data shows encouraging possibilities, several deficiencies in the design and implementation of studies generate doubts about the reliability of these outcomes. Given that embryo implantation is influenced by multiple factors (such as inflammatory and immune factors), maximizing the therapeutic effect of PRP requires individualized assessment based on each patient’s specific condition, thereby enabling more effective validation of PRP efficacy.

### 3.4. Safety evidence

PRP is obtained from the individual’s blood and entails the processing of its components during preparation, which may introduce potential risks for infection and immune responses. Additionally, several uncertainties remain about the long-term impacts of PRP on the health of both mothers and their children. Another important issue pertains to the need for regulatory and ethical guidelines, which require precise operational protocols, strict oversight and ethical standards to guarantee the safe and effective use of PRP for treating IUA. To comprehensively evaluate PRP’s safety in this context and address these issues, further research is required, including large-scale randomized controlled trials and systematic reviews, to enhance its practical applicability for IUA. During PRP preparation, inadequate aseptic techniques when handling blood components heighten contamination and infection risks. Research indicates that PRP shows considerable antimicrobial effects against a variety of microorganisms, such as Staphylococcus aureus, Neisseria gonorrhoeae and Group A Streptococcus, underscoring its promising role in preventing infections [55]. Therefore, to ensure patient safety and therapeutic efficacy, strict adherence to aseptic technique is imperative during PRP preparation and infusion.

Clinically applied PRP currently derives from autologous blood, thereby avoiding immunogenicity and transmission risks. Furthermore, based on outcomes from thousands of oral and maxillofacial surgery patients receiving postoperative PRP treatment, this therapy is considered safe [56]. More precisely, infections and other adverse events remain uncommon among patients. For intrauterine adhesions, PRP is a treatment option with a favourable safety profile, demonstrating only minor and transient adverse events in the short term. However, long-term follow-up data and precise risk stratification for high-risk populations remain relatively limited. The largest meta-analysis to date [17], which included 730 patients from 10 clinical studies, reported that three of these studies mentioned adverse events, with no observed occurrences of rash, fever, abdominal pain, abnormal uterine bleeding, or thrombosis [10,37,42]. The remaining studies did not describe any adverse events. In the meta-analysis conducted by Tang et al. (21 RCTs, 2406 patients), the safety profiles of various postoperative intervention protocols were systematically compared, including PRP ± balloon, amniotic membrane ± balloon, intrauterine device(IUD), sodium hyaluronate (HA), G-CSF, etc [57]. Among these, PRP, amniotic membrane and HA all require intrauterine procedures with similar operation-related risks (e.g. infection, perforation, albeit with extremely low incidence rates). IUD carries device-related risks (e.g. embedment, displacement)that are not associated with PRP. The amniotic membrane, being allogeneic in origin, theoretically carries a risk of immune rejection. Among all the compared interventions, PRP stands out as one of the options with the most well-defined safety profile. Its autologous origin fundamentally eliminates risks such as infection and immune rejection associated with allogeneic materials.

While the use of PRP monotherapy is beneficial in facilitating endometrial repair after IUA surgery, it is essential to acknowledge its shortcomings. The primary mechanism of PRP involves the stimulation of growth factors. Nevertheless, in situations characterized by intense adhesions or considerable injury to the endometrial basal layer, its ability to promote repair might be inadequate. Full restoration of standard endometrial function may not be achievable, and there is a potential for variable effectiveness. To address these issues, employing combined PRP therapies reveals notable benefits. As a result, integrated PRP treatment strategies are seen as a more progressive contemporary method.

### 3.5. Application advantages of autologous PRP

The core advantage of autologous PRP lies in its autologous origin, which avoids the immunological rejection reactions and disease transmission risks associated with allogeneic or xenogeneic products, thereby significantly enhancing treatment safety and patient acceptance. Nevertheless, the main drawbacks include a lack of adequate safety data (for instance, concerning its impact on endometriosis) and insufficient standardization in the preparation of PRP. This leads to variations in the quality and quantity of PRP products, ultimately affecting the achievement of optimal therapeutic results [58]. A meta-analysis involving ten clinical trials (with 730 participants) indicated that autologous PRP enhances menstrual flow volume(WMD = 2.96, 95% CI = 2.30–3.61; *p* < 0.001), days of menstruation(WMD = 1.13, 95% CI = 0.86–1.41; *p* < 0.001), boosts endometrial thickness(WMD = 0.79, 95% CI: 0.40–1.19; *p* < 0.001) and increases clinical pregnancy rates(OR = 1.82, 95% CI: 1.19–2.78; *p* = 0.006). However, this analysis found no significant differences in the recurrence rates of moderate-to-severe IUA, changes in FS scores, rates of miscarriage, or rates of live births [17]. Both autologous and allogeneic PRP showed favourable safety profiles, with few adverse events reported. While short-term studies suggest that PRP may enhance endometrial thickness and pregnancy outcomes, information regarding its long-term effects on reproductive results and maternal health is still insufficient. Therefore, longitudinal studies are necessary to assess the long-term safety and effectiveness of PRP, including its potential effects on future pregnancies and neonatal health [59].

As PRP is classified as ‘autologous cell therapy’, its regulatory pathways vary significantly worldwide, directly impacting the technology’s clinical application, research development and patient accessibility. In the United States and Australia, conventional PRP treatments are relatively easy to implement, while in China, they must be administered within registered medical institutions using approved preparation kits. These regulatory discrepancies have led to inconsistent PRP preparation protocols and quality control standards across countries, which is also one of the reasons for variations in PRP treatment research outcomes.

Although there are current prospective studies, the overall participant count is still comparatively low [42] and the durations of follow-up tend to be short. It is essential to conduct future extensive, long-term longitudinal investigations to better assess the safety and effectiveness of PRP in more substantial groups, especially regarding its impact on long-term reproductive results, pregnancy progression and neonatal well-being.

A critical factor contributing to the heterogeneity in clinical outcomes is the marked variability in PRP preparation protocols. Key parameters that differ across studies include: (1) Centrifugation protocols: variations in centrifugal force (ranging from 150 g to 1000 g), duration and the number of spins (single vs. double spin), which directly influence platelet yield and concentration; (2) platelet concentration: the final platelet count, often reported as a fold-increase over baseline (ranging from 2- to 8-times), lacks consensus on an optimal therapeutic range; (3) Activation method: PRP can be used in its non-activated (native) or activated form (using calcium chloride, thrombin, or physical methods like freeze-thawing), which significantly affects the kinetics and quantity of growth factor release and (4) leukocyte content: the presence or absence of leukocytes (leading to pure PRP vs. leukocyte-rich PRP) may influence the inflammatory milieu. The lack of standardized, internationally accepted guidelines for PRP preparation in reproductive medicine poses a major challenge for interpreting study results and hampers the translation of research findings into routine clinical practice.

## 4. Combined PRP therapy: synergistic mechanisms and clinical outcomes

**Figure 2.**
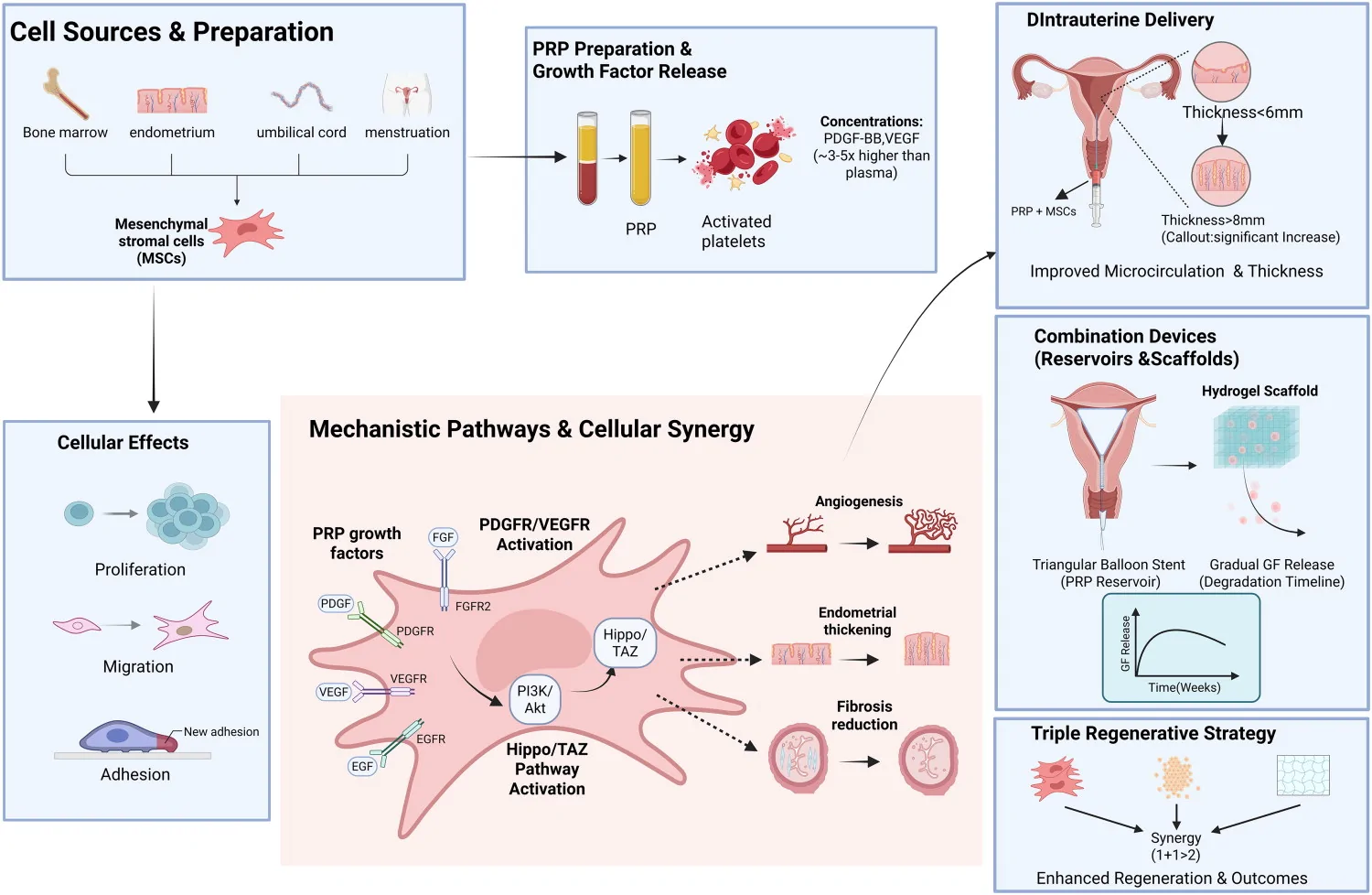
Synergistic mechanisms and clinical outcomes of PRP combination therapy.

For endometrial repair and regeneration, the ‘Triple Synergistic Therapy Strategy’ integrates MSCs, PRP and advanced delivery systems. This strategy aims to improve the endometrial microenvironment, promote angiogenesis and ultimately enhance clinical outcomes through the synergistic effects of multiple mechanisms (Figure 2).

This figure systematically illustrates a ‘triple synergistic therapeutic strategy’ for endometrial repair and regeneration, integrating cell therapy, PRP and advanced delivery systems. MSCs provide regeneration potential; PRP releases growth factors (PDGF, VEGF, etc.) to enhance cell behaviour. The scaffold enables localized sustained delivery. Mechanistically, it activates PDGFR/VEGFR, PI3K/Akt and Hippo/TAZ pathways to promote angiogenesis, reduce fibrosis and restore endometrial function, achieving a synergistic ‘1 + 1 > 2’ effect for treating intrauterine adhesions.

### 4.1. Synergistic application of PRP combined with MSCs

#### 4.1.1. Synergistic mechanisms

**Figure 3.**
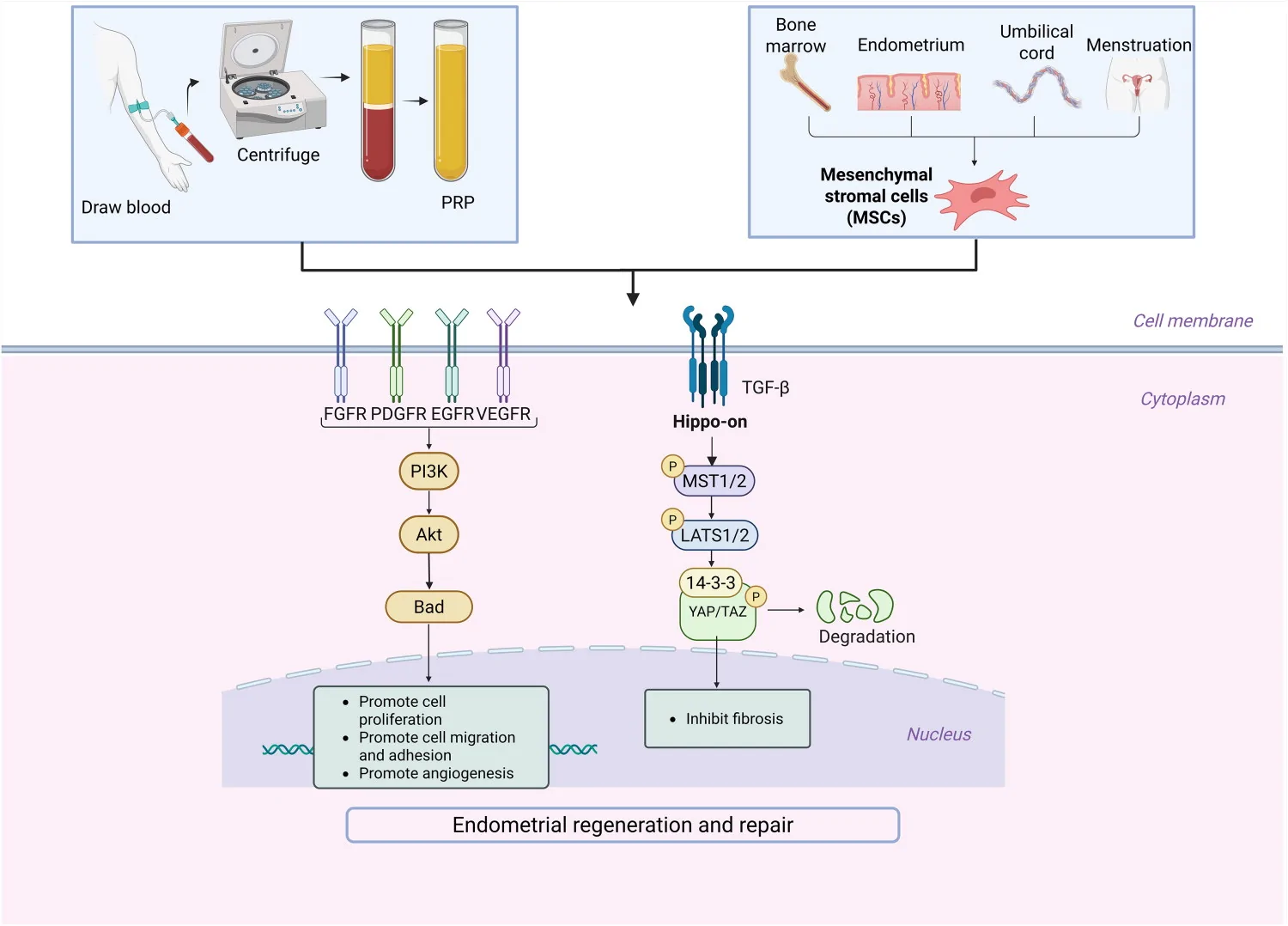
The synergistic mechanism of PRP and MSCs in promoting endometrial regeneration.

The combined effects of PRP and MSCs primarily manifest in enhancing cell proliferation, migration, angiogenesis and the activation of essential signalling pathways. Human MSCs are mainly derived from various tissues, including bone marrow, endometrium, umbilical cord, or menstrual blood and can be used for endometrial regeneration. Autologous PRP possesses attributes that facilitate cell growth and angiogenesis [49,60], effectively promoting the migration and adhesion of endometrial stromal cells *in vitro* [61]. This action is attributed to growth factors contained in platelet granules, which are released during platelet activation (Figure 3). Efendieva et al. undertook a comparative study of PDGF-BB and VEGF protein levels in PRP compared to conventional plasma from the same source, revealing that PRP had significantly elevated concentrations of both [62]. The study employed hysteroscopic-guided injection of PRP and endometrial cells suspended within it into the endometrium. Results demonstrated that PRP injection significantly improved endometrial thickness and local microcirculation (assessed *via* uterine spiral artery visualization rate). Furthermore, PRP injection supplemented with endometrial cells equally promoted endometrial thickening, with both approaches enhancing cellular proliferation and angiogenesis. However, a limitation of this study was the absence of comparative efficacy analysis between the two treatment modalities.

To obtain PRP, whole blood undergoes centrifugation. The growth factors present in PRP, including VEGF, PDGF, EGF and FGF, interact with MSCs sourced from bone marrow, endometrium, umbilical cord, or menstrual blood, thereby activating downstream signalling pathways such as PI3K/Akt and Hippo/TAZ. This combined effect facilitates an increase in cell proliferation, improves both cell migration and adhesion, stimulates the formation of new blood vessels and suppresses fibrosis, ultimately resulting in the regeneration and repair of the endometrium.

Wang et al. investigated the effects of PDGF and its combination with MSCs on the *in vitro* and *in vivo* regeneration of the endometrial epithelium [63]. *In vitro* experiments demonstrated that both MenSCs and PDGF alone significantly promoted cell proliferation, migration, invasion and neovascularization, with the combination yielding even more pronounced effects. This indicates a synergistic effect between MenSCs and PDGF in promoting cell proliferation, migration, invasion and angiogenesis. CD34 is considered the most sensitive marker for detecting microvascular formation. By assessing CD34 expression in endometrial tissue, the study revealed that both MenSCs and PDGF significantly promoted microvascular formation in endometrial epithelial cells, with a synergistic effect observed when used in combination. Furthermore, combined PDGF and MenSC intervention markedly enhanced Akt and Bad phosphorylation levels in endometrial tissue, with effects markedly superior to either PDGF or MenSC monotherapy. These findings indicate that the Akt and Bad signalling pathways play crucial roles in promoting epithelial regeneration, suggesting that the positive effects of PDGF and MenSCs on repairing surgery-induced endometrial injury partially depend on the activation of these pathways. The Hippo/TAZ signalling pathway constitutes a key mechanism through which EndoMSCs suppress endometrial stromal fibrosis [64]. Further research indicates EndoMSCs significantly accelerate endometrial injury repair *via* paracrine effects and Hippo pathway activation, with enhanced efficacy when combined with PRP [31]. A preliminary investigation conducted by Tandulwadkar and Karthick examined the combined effects of autologous bone marrow-derived stem cells (ABMDSCs) and PRP on ovarian rejuvenation [65]. The study found that autologous PRP facilitated the recruitment of stem cells to the site of injury and improved their response. This suggests that PRP boosts the bioactivity of stem cells by stimulating proliferation, migration and colony formation, ultimately enhancing regenerative potential.

#### 4.1.2. Clinical application advances

Clinical research demonstrates that the combination of autologous endometrial-derived MSCs with autologous PRP produces notable therapeutic benefits for individuals suffering from refractory thin endometrium [66]. After the intrauterine administration of these MSCs and PRP, patients showed a significant increase in endometrial thickness and a considerable enhancement in clinical pregnancy rates. In a cohort of 29 patients who underwent this treatment in conjunction with hormone replacement therapy to stimulate ovulation, 23 (79%) advanced to clinical pregnancy, with 10 (34.5%) resulting in live births. Tandulwadkar et al. documented a successful intervention for severe AS utilizing ABMDSCs in combination with PRP [67]. With the assistance of hysteroscopic guidance, the researchers injected ABMDSCs and PRP into the endometrial lining, leading to improved thickness and ultimately successful pregnancy outcomes.

### 4.2. Synergistic application of PRP combined with intrauterine stents

#### 4.2.1. Combined mechanism

Currently, there is a lack of studies investigating the mechanism behind the interaction of PRP with intrauterine stents. The synergistic effect of combining PRP with triangular balloon stents can be described as ‘1 + 1 > 2’. In this scenario, the stent serves as a ‘reservoir’ for PRP, with its mesh structure or surface facilitating the retention of PRP, which extends its duration within the uterine cavity and allows for a gradual release of growth factors instead of a rapid loss. Simultaneously, PRP provides the stent with ‘biological activity’: while the stent creates a physical framework, PRP infuses this framework with biological activity, thereby establishing a microenvironment conducive to cell migration, proliferation and differentiation. Together, these components work in harmony to enhance repair outcomes: the physical support offered by the stent reduces the risk of early adhesion recurrence, while the biological stimulation significantly fosters the functional regeneration of the endometrium. Ultimately, this collaboration aims to restore both the morphology of the uterine cavity and its reproductive functionality.

#### 4.2.2. Clinical outcomes

In a retrospective study by Peng et al. intrauterine infusion of PRP, intrauterine balloon placement and combined therapy were compared regarding their effects on AFS scores and chemical pregnancy rates [36]. Results demonstrated no statistically significant differences among the three groups. In Shen et al.’s study, patients undergoing IUA were divided into two groups: PRP combined with intrauterine-suitable balloon (ISB) and a control group receiving ISB alone [37]. Both groups showed a notable decrease in AFS scores after surgery, with the PRP group displaying a considerably larger median decrease compared to the control group (*p* < 0.05). Additionally, both groups exhibited significant improvements in Pictorial Blood-loss Assessment Chart (PBAC) scores when compared to preoperative values. However, the median rise in PBAC scores was considerably higher in the PRP group than in the control group (29 points vs 16 points, *p* < 0.001). In the PRP group, there was a significant postoperative increase in endometrial thickness compared to preoperative levels, while the control group did not present any significant variation. In contrast to earlier studies, Shen et al. utilized cervical balloons from the ISB system to extend PRP retention in the uterine cavity, which might contribute to improved PRP effectiveness for endometrial regeneration and wound healing. A systematic review and meta-analysis pooled data from 21 randomized controlled trials involving a total of 2,406 patients. The interventions included balloon, amniotic membrane, platelet-rich plasma, intrauterine device, hyaluronic acid, platelet-rich fibrin and granulocyte colony-stimulating factor. All of the following treatments – PRP+balloon, amniotic membrane + balloon, IUD+balloon, hyaluronic acid + balloon and granulocyte colony-stimulating factor (G-CSF)+balloon – were effective in reducing AFS scores. According to the SUCRA ranking, PRP+balloon demonstrated superior efficacy. In terms of improving clinical pregnancy rates, there was no statistically significant difference between PRP+balloon and amniotic membrane + balloon, IUD+balloon, or G-CSF+balloon. Based on SUCRA analysis, PRF, G-CSF+balloon and PRP+balloon may be better approaches for enhancing clinical pregnancy rates after IUA surgery. Regarding the recurrence rate of moderate-to-severe intrauterine adhesions, PRP+balloon, amniotic membrane + balloon and hyaluronic acid + balloon did not significantly reduce recurrence. SUCRA sequencing results indicated that PRP+balloon might be a more effective intervention. This meta-analysis suggests that platelet-rich plasma combined with balloon may currently be the most effective treatment option [57].

### 4.3. PRP combined with hydrogel

Hydrogels are polymeric substances characterized by a three-dimensional hydrophilic network architecture, which demonstrates a high capacity for adsorption and flexibility in response to environmental alterations [68]. They facilitate the efficient transport and release of drugs while also offering extended support to injured tissues. Hyaluronan (HA), an essential element of the ECM, promotes cell movement by modifying matrix assembly and creating porous formations [69]. Currently, crosslinked HA hydrogels available on the market (such as Gong’an Kang) are used clinically to manage IUA, acting as a physical barrier to curb recurrence. A study by Xie et al. introduced an innovative injectable, biodegradable hydrogel that functions effectively as a scaffold to sustain the release of growth factors from PRP while also acting as a physical barrier [70]. *In vivo* studies using a rat model of uterine adhesions showed that the administration of the PRP-loaded hydrogel through injection significantly lowered fibrosis and encouraged endometrial regeneration, thereby aiding in the restoration of fertility. Furthermore, Yu et al. created a new injectable hydrogel composed of PRP-loaded CL-PF127, which effectively inhibited endometrial fibrosis and stimulated angiogenesis in an SD rat model of uterine adhesion [71]. *In vitro* cellular investigations revealed that this hydrogel also alleviates inflammatory responses through the NF-κB signalling pathway. According to the research conducted by Chen et al. a dual-network hydrogel that combines commonly used hyaluronic acid (HA) with PRP was developed and utilized in patients suffering from moderate-to-severe uterine IUA [72]. They observed that, compared to the HA-only treatment group, dual-network hydrogel therapy significantly increased menstrual flow volume and endometrial blood perfusion, reduced IUA scores and elevated the number of endometrial glandular cells. Furthermore, this treatment markedly enhanced the expression levels of oestrogen receptor alpha (ERα), the cell proliferation marker (MKI67) and the platelet-derived endothelial cell adhesion molecule (CD31). The analysis of single-cell sequencing indicated a notable increase in the WNT signalling pathway’s activity within the endometrium after the application of dual-network hydrogel treatment. Immunohistochemistry validated that there was a heightened expression of SOX9 along with leucine-rich repeat-containing G protein-coupled receptor 5 (LGR5) in endometrial glands following treatment. Furthermore, *in vitro* studies showed that the dual-mesh hydrogel effectively stimulated the expression of both SOX9 and LGR5 in endometrial epithelial organoids. Measurements of swelling rates and *in vivo* degradation assessments demonstrated that the dual-mesh hydrogel exhibited enhanced stability when compared to pure HA and PRP, displaying a continuous, gradual, linear release of growth factors. These characteristics allow the dual-network hydrogel to improve the therapeutic effectiveness of PRP, support both repair and regeneration of the endometrium and successfully mitigate the recurrence of uterine adhesions.

### 4.4. Triple regeneration strategy

**Figure 4.**
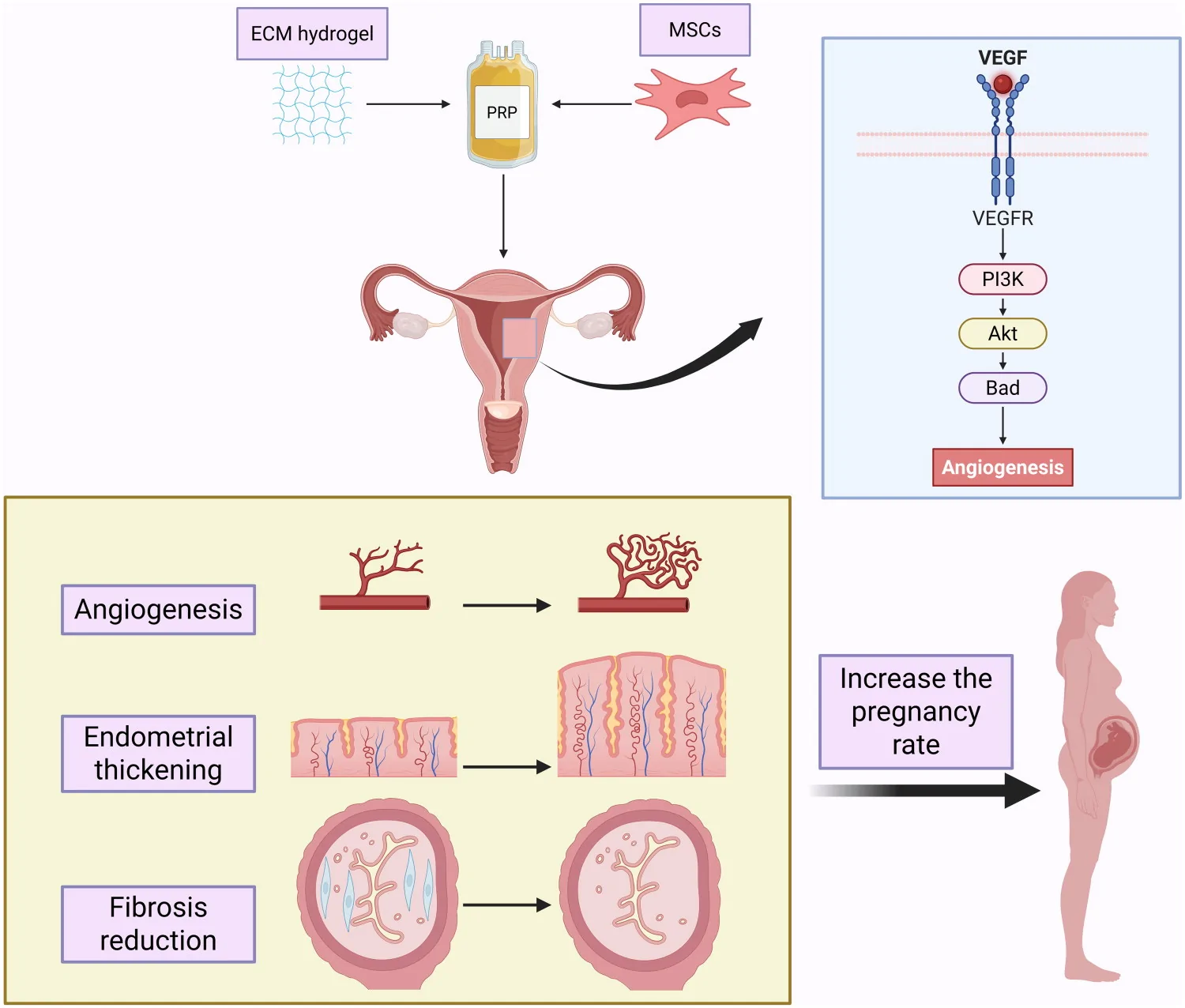
Schematic diagram of the ‘triple regeneration strategy’ for IUA.

Rodriguez-Eguren et al. provided a comprehensive overview of the biotechnological advancements in regenerative therapies aimed at addressing endometrial disorders, including IUA [18]. This evolution encompasses a range of treatments, from cell therapies to decellularized and bioengineered solutions, along with a transition from *in vitro* to *in vivo* and clinical applications. They introduced the concept of a ‘triple regenerative strategy’, which integrates MSCs, such as those sourced from bone marrow or umbilical cord, with therapeutic components derived from decellularized materials (e.g. PRP), all within ECM hydrogels. The implementation of this strategy is exemplified in the research conducted by Zheng et al. where an injectable hydrogel was formulated [19]. This self-healing, antioxidant-enriched hydrogel is composed of thiolated hyaluronic acid (tHA) and thiolated chitosan (tChi), specifically engineered to encapsulate PRP alongside adipose-derived stem cells. Employing the ‘triple regenerative strategy’ resulted in notable improvements in endometrial regeneration within a murine model exhibiting thin endometrium, as it triggered the VEGF/AKT/BAD signalling pathway, thereby facilitating endothelial angiogenesis. These enhancements were evidenced by increases in endometrial thickness and a decrease in fibrosis, ultimately leading to improved endometrial receptivity and higher pregnancy rates. With the continuous advancements in biotechnology and the enhancement of preclinical modelling research, this study establishes a crucial groundwork for the formulation of personalized therapeutic interventions for infertility-related endometrial issues, including IUA (Figure 4).

The ‘triple regeneration strategy’ integrates MSCs and PRP into an ECM hydrogel. This composite system activates the VEGF/AKT/BAD signalling pathway in endothelial cells. As a result, it promotes angiogenesis, increases endometrial thickness and reduces fibrosis. Ultimately, these changes enhance endometrial receptivity and improve pregnancy outcomes.

The integration of PRP, stem cells and hyaluronic acid (HA) signifies a pivotal shift in intrauterine adhesion treatment from ‘single intervention’ to ‘tissue microenvironment reconstruction’. Among these, hyaluronic acid holds the highest evidence level (Level 1) and has been widely applied in clinical practice as a medical device. The meta-analysis by Luo et al. which included 16 RCTs with a sample size of 2,359 cases, demonstrated that HA can safely and effectively reduce the incidence of moderate to severe intrauterine adhesions. Their trial sequential analysis confirmed that no further clinical trials are needed to validate these findings [73]. The evidence level for PRP is moderate (Level 2), with multiple meta-analyses confirming its effectiveness in improving endometrial thickness and clinical pregnancy rates [17]. However, regarding the ultimate outcome measure of live birth rate, the evidence remains insufficient, warranting a weak recommendation grade. The evidence level for stem cells is relatively low (Level 3–4). Although phase I clinical trials have been published, they are all small-scale exploratory studies [74]. The 2025 autologous endometrial stem cell study included only 15 patients [75], far below the evidence volume required for routine clinical application. The integrated evidence level for HA+PRP+stem cells is very low (preclinical), currently limited to animal experiments, with no human clinical trial evidence supporting its safety and efficacy [19]. Currently, any treatment regimen involving stem cells remains in the early research stage and is limited to clinical trials. It is strongly advised against routine clinical application. The combination of HA + PRP + stem cells only has supporting evidence from animal studies, with no human safety data available and is still in the preclinical research phase [19]. It is strictly prohibited to promote this as a routine treatment. To advance the integrated strategy of HA + PRP + stem cells from a ‘theoretical model’ to ‘clinical application’, rigorous clinical trials must be designed for validation, adhering to the principles of ‘ethical registration, phased implementation, strict oversight and long-term monitoring’. The clinical characteristics and efficacy outcomes of different PRP combined therapy regimens for moderate to severe IUA are summarized in Table 4.

**Table 4.** Comparison of combined treatment methods with platelet-rich plasma.

Study ID	Country	Study design	Population	Sample size	Outcome measure	Main discovery
Peng et al. [36]	China	Retrospective	Moderate to severe IUA	PRP: 28 Balloon: 22 PRP+Balloon: 20	1. AFS score 2. Rates of chemical pregnancy	None were statistically significant
Shen et al. [37]	China	RCT	Moderate to severe IUA	PRP+Balloon: 63 Balloon: 60	1. AFS score 2. PBAC score 3. Endometrial thickness	All were statistically significant
Efendieva et al. [60]	Russia	Pilot randomized study	TE	Conservative treatment: 30 PRP: 42 Conservative treatment + PRP: 38 Autologous endometrial cells suspended in PRP: 5	1. Endometrial thickness 2. Spiral artery imaging rate of the uterus 3. Clinical pregnancy rate 4. Live birth rate	Both PRP and PRP supplemented with endometrial cells can significantly improve endometrial thickness and local microcirculation
Chen et al. [72]	China	Prospective study	Moderate to severe IUA	PRP double-network hydrogel (DN hydrogel group): 11 Cross-linked sodium hyaluronate gel (HA group): 8	1. PBAC score 2. AFS score 3. Endometrial thickness 4. Endometrial blood flow	PRP double-network hydrogel improves PBAC score and increases endometrial blood flow

*Note:* TE: thin endometrium; RCT: randomized controlled trial; PBAC: Pictorial Blood-loss Assessment Chart; AFS: American Fertility Society.

## 5. Limitations

This review has several limitations that should be acknowledged. Firstly, as a narrative review, it is inherently subjective and does not employ a systematic search strategy or formal quality assessment of included studies, which may introduce selection bias. Secondly, the available evidence base is itself limited by the small sample sizes of most clinical trials, the lack of long-term follow-up data and the scarcity of well-designed, double-blind, randomized controlled trials, particularly those with live birth as a primary endpoint. Thirdly, the significant heterogeneity in PRP preparation and administration protocols across studies severely limits the comparability of findings and precludes the formulation of definitive clinical recommendations. Finally, the majority of mechanistic insights are derived from *in vitro* and animal models, which may not fully recapitulate the complex pathophysiology of IUA in humans. These limitations underscore the need for cautious interpretation of the current evidence and highlight the critical areas for future research.

## 6. Conclusions and future directions

In summary, PRP primarily reduces intrauterine adhesion recurrence and improves endometrial receptivity, thereby enhancing clinical outcomes, through mechanisms such as promoting endometrial cell proliferation, inhibiting endometrial fibrosis, regulating the proliferation and migration of endometrial mesenchymal stem cells, stimulating angiogenesis and modulating inflammatory responses. However, the specific signalling pathways involved remain unclear. The clinical efficacy of PRP shows some variability across studies. The meta-analysis by Tang et al. currently represents the highest-quality evidence in the field of PRP treatment for intrauterine adhesions, demonstrating that PRP significantly increases endometrial thickness, improves menstrual volume and enhances clinical pregnancy rates (OR 1.82) [17]. However, insufficient evidence exists regarding its effects on live birth rates, miscarriage rates and recurrence rates of moderate-to-severe adhesions.

The RCT by Aghajanova et al. demonstrated that PRP perfusion did not significantly improve endometrial thickness or pregnancy rates in patients with moderate-to-severe adhesions [42]. This remains the only RCT specifically targeting moderate-to-severe adhesions, and although the sample size was small (30 cases), it also suggests that PRP may have limited efficacy in severe cases. Based on relevant research findings, there is substantial evidence for improvement in endometrial thickness and menstrual volume, moderate evidence for enhanced clinical pregnancy rates, but insufficient evidence regarding live birth rates and long-term safety. PRP treatment can be considered for mild-to-moderate adhesions, patients who have failed conventional treatments, thin endometrium and recurrent implantation failure(RIF), but is not recommended for primary preventive use. Prior to application, thorough physician-patient communication must be conducted to clarify the current limitations of evidence: PRP can improve endometrial thickness and clinical pregnancy rates, but its impact on live birth rates has not been confirmed [17]. Additionally, as it is mostly a self-pay service, patients’ financial conditions should be comprehensively considered. The current heterogeneity in PRP treatment efficacy and evidence gaps are primarily attributed to the following factors: First, patient selection – there is insufficient evidence regarding efficacy differences based on adhesion severity stratification (mild-moderate vs. severe) and the influences of aetiology specificity, treatment history, age and other factors remain unknown. Second, standardization of preparation – there is no consensus on the optimal platelet concentration (ranging from 2 to 8 times), activation method (pre-activated vs. non-activated), administration timing (post-menstruation or mid-cycle), or administration frequency (single vs. multiple doses). Third, outcome measures: There is a lack of high-quality evidence regarding key issues such as live birth rate, miscarriage rate, moderate-to-severe recurrence rate, long-term safety (offspring health) and cost-effectiveness ratio. PRP therapy remains in the clinical exploratory stage.

Autologous PRP is subject to relatively lenient regulation (classified as a ‘361 Product’ in the U.S. and as a Category III medical technology requiring record-filing in China) and can be administered in registered institutions, though it is mostly self-funded and not covered by medical insurance. Allogeneic PRP (derived from umbilical cord blood) has higher growth factor concentrations and allows standardized preparation but is regulated as a drug or biological product, requiring a complex approval process. Currently, its use is limited to clinical trials. To clarify the clinical efficacy of PRP in patients with intrauterine adhesions, it is imperative to conduct multicentre, randomized, controlled, double-blind clinical trials with live birth rate as the primary endpoint. These trials should focus on moderate-to-severe adhesion cases (AFS score ≥ 5), adopt a standardized PRP preparation protocol and require a sample size of 200–250 cases or more per group. Concurrently, it is essential to investigate the therapeutic dosage of PRP (e.g. optimal platelet concentration, single vs. multiple doses, activated vs. non-activated) and gather real-world evidence (through multicentre prospective cohort studies and long-term follow-up registries).

Future research should elucidate the precise mechanisms by which PRP affects the endometrium and explore optimal combination therapies that may enhance its efficacy. Furthermore, the treatment of intrauterine adhesions should be individualized, and the therapeutic approach can be adjusted based on factors such as the severity of adhesions, endometrial thickness, uterine cavity size and the patient’s age.

In conclusion, PRP represents a promising, safe and biologically plausible adjuvant therapy for IUA, with evidence supporting its role in improving endometrial thickness and menstrual patterns. However, its definitive effectiveness in enhancing live birth rates and preventing adhesion recurrence remains unproven due to the current low-to-moderate quality of evidence. The widespread adoption of PRP therapy is significantly hampered by the lack of standardized preparation protocols, the variability in clinical application and unresolved cost-effectiveness concerns. Therefore, while PRP should not yet be considered a standard of care, it may be considered as a therapeutic option for select patients with refractory thin endometrium or recurrent implantation failure, ideally within the context of well-designed clinical trials. Future research must prioritize the establishment of consensus guidelines for PRP preparation and the conduct of large, multicentre, randomized controlled trials to conclusively determine its clinical value and optimize patient selection.

## Data Availability

Data availability is not applicable to this article as no new data were created or analysed in this study.
